# Effect of Diacylglycerol Crystallization on W/O/W Emulsion Stability, Controlled Release Properties and In Vitro Digestibility

**DOI:** 10.3390/foods12244431

**Published:** 2023-12-10

**Authors:** Chaoying Qiu, Yingwei Liu, Canfeng Chen, Yee Ying Lee, Yong Wang

**Affiliations:** 1 JNU-UPM International Joint Laboratory on Plant Oil Processing and Safety, Department of Food Science and Engineering, Jinan University, Guangzhou 510632, China; tcyqiu@jnu.edu.cn (C.Q.); 13178571129@163.com (C.C.); 2 Hunan Edible Fungi Institute, Changsha 410013, China; 18774943216@163.com; 3 School of Science, Monash University Malaysia, Bandar Sunway 47500, Selangor, Malaysia; lee.yeeying@monash.edu

**Keywords:** W/O/W emulsion, diacylglycerol, interfacial crystallization, osmotic pressure, in vitro release profiles

## Abstract

Water-in-oil-in-water (W/O/W) emulsions with high-melting diacylglycerol (DAG) crystals incorporated in the oil droplets were fabricated and the compositions were optimized to achieve the best physical stability. The stability against osmotic pressure, encapsulation efficiency and in vitro release profiles of both water- and oil-soluble bioactives were investigated. The presence of interfacial crystallized DAG shells increased the emulsion stability by reducing the swelling and shrinkage of emulsions against osmotic pressure and heating treatment. DAG crystals located at the inner water/oil (W_1_/O) interface and the gelation of the inner phase by gelatin helped reduce the oil droplet size and slow down the salt release rate. The DAG and gelatin-contained double emulsion showed improved encapsulation efficiency of bioactives, especially for the epigallocatechin gallate (EGCG) during storage. The double emulsions with DAG had a lower digestion rate but higher bioaccessibility of EGCG and curcumin after in vitro digestion. DAG-stabilized double emulsions with a gelled inner phase thus can be applied as controlled delivery systems for bioactives by forming robust interfacial crystalline shells.

## 1. Introduction

Water-in-oil-in-water (W/O/W) double emulsions can be applied in the field of low-fat food such as sauces, dressings or creams, or be used as vehicles for delivery of functional nutrition compounds including salt, iron [1], quercetin [2], peptides [3], probiotics [4], curcumin, luteins, etc., in dietary products [5]. The existence of two different aqueous phases and one oil phase were efficient to control the release of hydrophilic ingredients and behave as an effective strategy to co-deliver oil- and water-soluble compounds with synergistic functions [6]. For the fabrication process, two steps of emulsification are generally required by first creating the W_1_/O emulsion before subsequent dissemination of the external phase W_2_ in the second stage, during which both hydrophilic and lipophilic emulsifiers are required [7]. Therefore, the characteristics of W/O emulsion are critical for the W/O/W emulsion stability. Compared with O/W emulsion, W/O emulsion is more susceptible to sedimentation, flocculation and coalescence due to the low thermodynamic stability of the oil droplet suspensions [8]. Thus increasing the rigidity of oil film and the physical stability of oil droplets are critical for the fabrication of W/O/W emulsion.

In the double emulsions, the W_1_/O interface was normally stabilized by low-HLB emulsifiers including PGPR (polyglycerol polyricinoleate), soybean lecithin, and Span 60/80. Proteins and polysaccharides were utilized as water-soluble emulsifiers for the O/W_2_ interface. Electrolyte compounds, such as NaCl are occasionally added to enhance the interfacial layer stability [8,9]. Studies have focused on the modulation of osmotic regulation by quantification of the transport kinetics [10]. Nonetheless, since the single emulsion in double emulsions is linked by the same oil phase and with low thermodynamic stability, the double emulsions are liable to diffusion and coalescence when subjected to environmental-condition changes. The shrinkage and swelling of the inner aqueous droplets easily occur by altering the osmotic pressure. To improve the stability of double emulsion, strategies such as internal phase gelation using polysaccharides or proteins, and fabrication of colloidal particles such as Pickering stabilizers were applied to impart the emulsions with a higher resistance to coalescence [11]. Colloidal particles such as zein, gliadin and pectin-BSA have been utilized as outer water phase stabilizers for W/O/W emulsions [12,13]. Colloidal particles can form a rigid particle layer and reduce the film drainage. Nonetheless, the types of oil-soluble lipid particles being able to stabilize the inner water phase are rarely reported. 

One strategy to stabilize the W/O interfaces is through using fat crystals such as waxes, hydrogenated soybean oil [14], fatty alcohols and monoglycerides [15], which can create a solidified structure acting as a physical barrier that can inhibit oil and water diffusion [10,16]. The stability of encapsulated bioactives was thus enhanced because a solidified lipid phase can slow down the diffusion of the compounds liable to chemical degradation. Nonetheless, the above systems still face some defects such as a high usage of solid lipid content and low storage stability of double emulsion [16]. In some cases, the existence of fat crystals in a dispersed oil phase could be a source of instability for O/W emulsions when piercing neighboring droplets, thus causing aggregation and coalescence [16]. In our previous research, a functional lipid diacylglycerol (DAG) was proved to be an efficient Pickering stabilizer by anchoring at the water–oil interface and forming a crystal network to stabilize the W/O emulsions. It is well known for its beneficial health attributes including reducing postprandial lipids and decreasing total cholesterol in serum [9]. In addition, DAG crystals showed a synergistic effect with PGPR in stabilizing high internal phase (>75% *v*/*v*) W/O emulsion (HIPE), which enabled the incorporation of a more internal water phase [9]. Moreover, the thickening polysaccharide presence in the water phase also improved the long-term stability of W/O emulsions which can better resist water droplets’ coalescence and have a higher retention ability for bioactives [9]. The crystallized lipids impart novel interfacial properties and influence the structural and physical stability of the double emulsions [17], which provide a sustained release of bioactives [10,18]. Up to now, although the W/O emulsions stabilized by lipid crystals and their in vitro release profiles have been reported, little information can be obtained on the DAG-stabilized W/O/W emulsions and the behavior of emulsions in the simulated gastrointestinal tract is still unknown.

Generally, during the digestion process of W/O/W emulsions, the release of internal W_1_ phase and intestinal absorption of bioactive compounds are required for nutritional purposes. Curcumin is a traditional hydrophobic nutraceutical with a number of beneficial biological benefits such as antitumor activity, anti-inflammatory activity and antioxidant activity [12]. Epigallocatechin gallate (EGCG) is extracted from green tea polyphenols and provides strong antioxidant activity which can protect against cardiovascular and other chronic diseases due to numerous phenolic hydroxyl groups in the molecular structure. Both of these compounds have low solubility, stability and oral bioavailability because of their rapid metabolism in gastrointestinal fluids and poor membrane permeability [19]. By incorporating into W/O/W emulsion, the compounds can be protected against undesirable gastrointestinal reactions. In addition, research has revealed that the W/O/W emulsion could enhance the anticancer activity of EGCG and curcumin, and their synergistic effect is advantageous for their application [20].

In this study, W/O/W double emulsions were prepared using high-melting DAG and gelatin to form a stable solid crystal layer around the inner water droplet surface and gelled internal water phase, respectively. The objective was to explore the DAG crystals formed in the lipid matrix and internal phase gelation on the physical stability and sustained release of hydrophilic and hydrophobic cargos in the simulated gastrointestinal tract (GIT). This study offers novel strategies for the rational design of double emulsions to co-deliver synergistic bioactive compounds.

## 2. Materials and Methods

### 2.1. Materials 

Sunflower oil (SO) was obtained from a grocery store. PGPR was kindly provided by Kerry Group Business Services (ASPAC) Sdn Bhd, Malaysia. Curcumin and EGCG (>95% purity) were bought from Yuanye Bio-Technology Co., Ltd. (Shanghai, China). Sodium caseinate (from milk), Nile Red (72485), Nile Blue A (N0766), porcine gelatin, porcine pancreatin (4 × USP), pepsin, mucin and bile extract (bile salt content ~49 wt.%) were bought from Sigma-Aldrich (St. Louis, MO, USA). Novozyme 435 was obtained from Novozyme (China) Biotechnology Co., Ltd. (Guangzhou, China). 

### 2.2. DAG Synthesis

DAG (66.34% DAG, *w*/*w* and 31.86% TAG, *w*/*w*) was synthesized through enzymatic esterification of glycerol and stearic acid with molecular distillation [21]. Stearic acid was mixed with glycerol (molar ratio of 2.25:1) and stirred with a magnetic stirrer (200 rpm at 70 °C) for 10 min in a round-bottom flask. Afterward, Novozyme 435 was added with an esterification process for 5 h (5 wt.% of total substrates at 0.1 MPa). The crude DAG was then purified by molecular distillation equipment MD III (Foshan Handway Technology Co., Ltd., Foshan, China) at an evaporation temperature of 220 °C and pressure of 0.1 Pa. 

### 2.3. Fabrication of W/O/W Emulsion

W/O/W emulsions were fabricated as reported elsewhere with slight modifications [22]. The effect of PGPR concentration (1–5%, *w*/*w* of total lipid phases) and the W_1_/O ratio (3:7, 5:5, 7:3, *w*/*w*) on the characteristics of W/O/W double emulsions were compared. The inner phase (W_1_) consisted of MgCl_2_ (0.1, 0.5 and 1 M) and gelatin in aqueous solution. Lipid phases were prepared by adding 1% (*w*/*v*) PGPR and 6% (*w*/*v*) DAG to SO, stirred for 15 min at 80 °C. The inner aqueous phase (W_1_) (85 °C) was then added dropwise into the lipid phase (SO) with stirring at 800 rpm for 10 min at 80 °C. The mixtures were homogenized using a high-shear homogenizer (IKA T25-Digital Ultra Turrax, Staufen, Germany) at 15,000 rpm for 6 min to obtain the primary W_1_/O emulsions. 

The external phase (W_2_) was prepared by dissolving 0.3 M glucose, 1.25% (*w*/*w*) sodium caseinate as a hydrophilic emulsifier, and 0.5% (*w*/*w*) xanthan gum, and preheated to 80 °C in a water bath. Double emulsions were prepared by blending W_1_/O emulsion with W_2_ at a ratio of 10:20 (*w*/*w*) using the same homogenizer at 15,000 rpm for 2 min at 80 °C. The freshly made W/O/W emulsions were quickly cooled to 25 °C under continuous stirring using the magnetic stirrer (500 rpm) in an ice bath to allow lipid crystallization. The double emulsions indicated by SO (the oil phase that only contains sunflower oil), DAG-SO (that oil phase that contains sunflower oil and DAG) and DAG-SO (the inner water phase that contains gelatin) were then stored at 25 °C for analysis.

#### Effect of Osmotic Stress on the Stability of Double Emulsion

Initially, the internal MgCl_2_ concentration in the W_1_ phase was 0.1 M, and the glucose concentration in the W_2_ phase was 0.3 M to put the emulsions under an osmotic equilibrium. The emulsions were then diluted by distilled water or glucose solutions to obtain W_2_ phases with 0.1, 0.3 and 0.6 M glucose. The emulsions were then stored at 25 °C for further analysis.

### 2.4. Microscopy Analysis

The freshly prepared emulsions were observed at room temperature using a polarized light microscope (Leica DM2700P, Wetzlar, Germany) in brightfield light modes. A slide with the W/O/W emulsion was placed inside a covered heating stage (Linkam LTS120, Tadworth, Surrey, UK) after equilibrating at 30, 40, 50, 60, 70 and 80 °C for 20 min, respectively, and the images were captured at 400× magnification. 

### 2.5. Confocal Laser Light Scanning Microscopy (CLSM)

For CLSM analysis, the oil phases, DAG crystals and proteins were stained by Nile Red (1 mg/mL) and Nile Blue A (1 mg/mL), respectively. The stained solution (20 μL) was placed on a slide and after the evaporation of acetone, samples were put into a sample holder and equilibrated to allow diffusion of Nile Red and Nile Blue A into emulsion. Pictures were captured using a Zeiss LSM 880 confocal laser scanning microscope (Carl Zeiss Inc., Oberkochen, Germany) at excitation/emission wavelengths of 543/605 nm and 633/697 nm for Nile Red and Nile Blue A, respectively.

### 2.6. Viscosity Measurement

The viscosity properties of W/O/W emulsions were measured using a Kinexus Pro rheometer (Malvern Instruments Ltd., Malvern, UK) with a Peltier system for controlling temperature and a parallel plate of 40 mm diameter at 5 °C. The geometry gap was set at 0.5 mm. The measurements were carried out in a shear rate range of 1–100 s^−1^.

### 2.7. Droplet Size Analysis

The droplet size distributions and mean droplet size of the emulsions were evaluated using a laser diffraction particle size analyzer (SALD-2300, Shimadzu, Kyoto, Japan). The emulsion was diluted by distilled water to a droplet concentration of 0.005% (*v*/*v*) to reduce multiple scattering effects [23]. 

### 2.8. Salt Release Behavior

The release profile of Mg^2+^ from the W_1_ phase in double emulsions was investigated using a conductivity meter (ORION-5-STAR, Thermo Scientific, Beverly, MA, USA). The probe was dipped into distilled water (100 mL) and then 5 g emulsion was added with stirring at 100 rpm. The release of Mg^2+^ by changing temperature was examined by heating the system to 80 °C on a stirring hotplate to observe the stabilization effect of fat crystals and measuring changes in conductivity. The release rate was calculated using the following equation:

(1) $$release(\%)=\frac{R_{t}}{R_{s}}\times 100\%$$

where $R_{s}$ is the total ion concentration embedded in the W_1_ phase, and $R_{t}$ is the magnesium ion concentration in the inner water phase at a specific time [24,25].

### 2.9. Encapsulation Efficiency of EGCG and Curcumin

The emulsions were diluted by an equal amount of medium with 25% ethanol and 0.5% Tween 80. Then, the emulsions were centrifuged at 4000 rpm for 10 min. The bottom solution with nonencapsulated EGCG and curcumin was taken for HPLC analysis and UV-Vis spectrophotometry, respectively. The encapsulation efficiency (EE) was calculated as the fraction of EGCG or curcumin that remained in the W_1_ or the oil phase in the W/O/W emulsion and expressed as

(2) $$EE\left(\%\right)=100\times (A_{total}-A_{outer})/A_{total}$$

Here, $A_{total}$ is the initial EGCG or curcumin amount added into the W_1_ or oil phase, and $A_{outer}$ is the encapsulated EGCG or curcumin amount existed in the W_2_ phase [13].

### 2.10. In Vitro Digestion Profile of Emulsions

The gastrointestinal (GIT) digestion model was carried out according to a previous study [26]. W/O/W emulsion (20 mL) was mixed with 20 mL of artificial saliva solution which contained 15 mg/mL porcine gastric mucin (type II). Then, the mixture was incubated at 37 °C for 10 min. Afterwards, 25 mL of oral fluid was mixed with 25 mL of gastric fluid solution with the addition of 1.6 mg/mL pepsin. The mixture was incubated at 37 °C for 2 h at pH 2. Following this, 30 mL of gastric fluid was then mixed with 1.5 mL of simulated intestine fluid and 3.5 mL of bile salt (pH adjusted to 7). Subsequently, lipase (2.5 mL, 1.6 mg/mL) was added to initiate the digestion and the pH was controlled with an automatic titration unit (Metrohm USA, Inc. Westbuy, NY, USA) at 37 °C. Then, 0.1 mM NaOH was titrated into the mixture to maintain the pH and incubated for 2 h. The water dispersion containing EGCG and oil dispersion containing curcumin with the same bioactive amounts were also digested according to the same procedure as the control. The FFA release was calculated according to the titration curves based on the following equation [27]:

(3) $$FFA\left(\%\right)=100\times \frac{V_{NaOH}\times C_{NaOH}\times M_{Lipid}}{2W_{Lipid}}\times 100\%$$

where $V_{NaOH}$ and $C_{NaOH}$ are the volume and molarity of the added NaOH solution, respectively, and $M_{Lipid}$ and $W_{Lipid}$ are the molecular weight (875 g/mol) and weight of sunflower oil, respectively.

### 2.11. Bioaccessibility Calculation

The digesta after digestion were collected and cooled in ice water, then centrifuged at 15,000 rpm (4 °C) for 30 min. The middle layer as micellar fraction was obtained by carefully pipetting with a dropper, and the contents of EGCG and curcumin in the micelle phase were determined. The bioaccessibility of EGCG and curcumin was calculated according to the following equation [26]:

(4) $$Bioaccessibility\left(\%\right)=100\times A_{s}/A_{initial}$$

Here, $A_{s}$ is the EGCG or curcumin amount in the micellar fraction, and $A_{initial}$ is the initial EGCG or curcumin amount in the digestion system.

### 2.12. EGCG and Curcumin Determination

The EGCG was determined using HPLC performed with LC 20AD (SHIMADZU, Tokyo, Japan) using a Diamonsil C18 column (5 μm, 150 × 4:6 mm) at 30 °C. A linear gradient elution program was referred to a previous study [28] with modification performed as follows: the injection volume was 10 μL and the mobile phase was 0.4% (*v*/*v*) water–formic acid (A) and acetonitrile (B) by firstly using 10–25% (*v*/*v*) B from 0 to 12 min, linear gradient to 10% (*v*/*v*) B from 12 to 14 min and then kept at 10% (*v*/*v*) B for 6 min. The flow rate was 1.0 mL/min and total run time was 20 min. The compounds were then detected at 280 nm and quantified based on the calibration curve of EGCG (Y = 3836.9X − 28,244, R^2^ = 0.9967). The curcumin concentration (mg/mL) was determined with a UV-Vis spectrophotometer at 425 nm using a calibration curve (Y = 0.0859X − 0.0144, R^2^ = 0.998).

### 2.13. Statistical Analysis

One-way analysis of variance (ANOVA) was carried out using SPSS 16.0 statistical software (SPSS Inc., Chicago, IL, USA). Statistical differences were considered as significant with a *p* value < 0.05 using Duncan’s new multiple-range post hoc test.

## 3. Results and Discussion

### 3.1. Characteristics of W/O/W Emulsion

#### 3.1.1. Effect of PGPR Concentration

**Figure 1:**
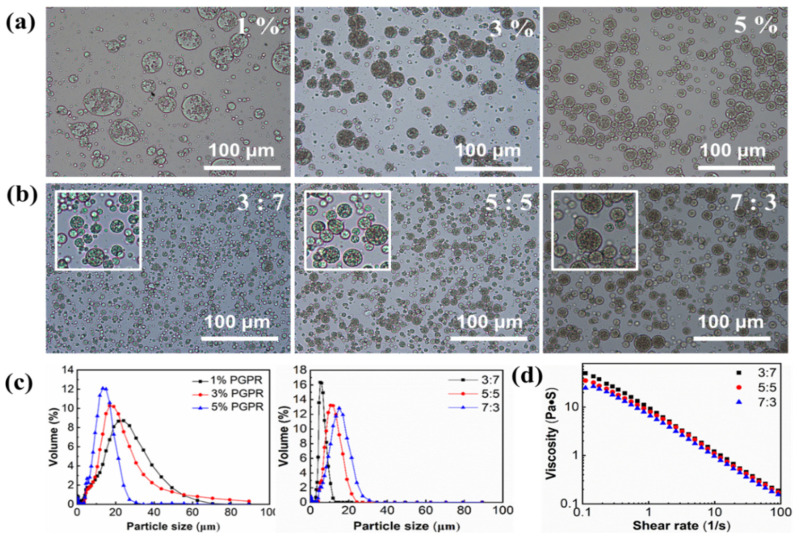
Optical micrograph of W/O/W emulsions containing different PGPR concentrations (1−5%, *w*/*w*) (**a**) and different W_1_: O ratios (**b**). The white frame in indicates the magnified area of images. Particle size distributions (**c**) and viscosity (**d**) of DAG-based W/O/W emulsion.

PGPR is the most commonly used stabilizer with a low HLB value (≈0.6) in W/O emulsions and the interaction of lipophilic emulsifiers with fat crystals would alter their abilities to stabilize double emulsions. We have proved that 6 wt.% DAG was a minimum concentration that can form stable W/O emulsion with an internal phase of 60% (*v*/*v*) in a previous study [9]. Herein, the influence of PGPR concentration on the physical stability of the double emulsion was investigated. The light microscopy images of the emulsions prepared using 6 wt.% DAG and a three-phase ratio (W_1_:O:W_2_) of 5:5:40 were obtained. As shown in Figure 1a, the microstructures of W/O/W emulsions with different PGPR levels varied greatly, although there is no difference in the visual appearance of the W/O/W emulsions ( When the PGPR concentration was 1 wt.%, irregular large water droplets were noticeable inside the W/O droplets, indicating that the PGPR amount was insufficient to stabilize the inner phase. In contrast, the PGPR of 3−5 wt.% can facilitate the formation of uniformly distributed water droplets into the oil phase. Furthermore, with increasing PGPR ratio, more emulsion droplets can be formed. The droplet size of emulsion is a critical parameter for determining the stability since large droplet size tends to cause coalescence, flocculation or Ostwald ripening of droplets [29]. As shown in Figure 1c and Table 1, all the emulsions showed a single peak with significant differences in droplet size of 14.71 ± 0.40, 13.63 ± 0.42 and 10.53 ± 0.28 µm, respectively, with 1, 3 and 5 wt.% PGPR (*p* < 0.05). This result was consistent with that of Tamnak and Mirhosseini [30], who reported that a PGPR concentration of 5 wt.% facilitated the formation of W/O/W emulsions with small droplet sizes. Therefore, 5 wt.% PGPR was chosen for stabilizing W/O emulsion in the following study. 

The effect of W_1_:O ratio is crucial for the physical stability of W/O/W emulsion. The microscope images and mean particle diameters of DAG-based W/O/W emulsions with different W_1_:O ratios were presented in Figure 1c and Table 2. All the emulsions showed unimodal size distribution, and the droplet size of the emulsions with W_1_:O ratios of 3:7, 5:5 and 7:3 were 5.53 ± 0.12, 9.86 ± 0.15 and 10.86 ± 0.30 µm, respectively. Overall, the droplet size was smaller than the previous report (20~40 µm) by using other lipid crystals [30], which indicated the good emulsifying properties of the DAG stabilizer and the high physical stability of double emulsions. The increased internal water phase caused an increase in the droplet size of the W_1_/O emulsions, which could account for the increased droplet sizes in the W/O/W emulsion. Considering the similar droplet size of emulsions with W_1_:O ratios of 5:5 and 7:3, and in order to incorporate a higher amount of water-soluble active agents in the inner phase, a W_1_:O ratio of 7:3 was chosen in the subsequent experiments. The W/O/W emulsions can retain stability with no visible phase separation for at least 2 months at room temperature.

#### 3.1.2. Effect of Salt Concentration

**Figure 2:**
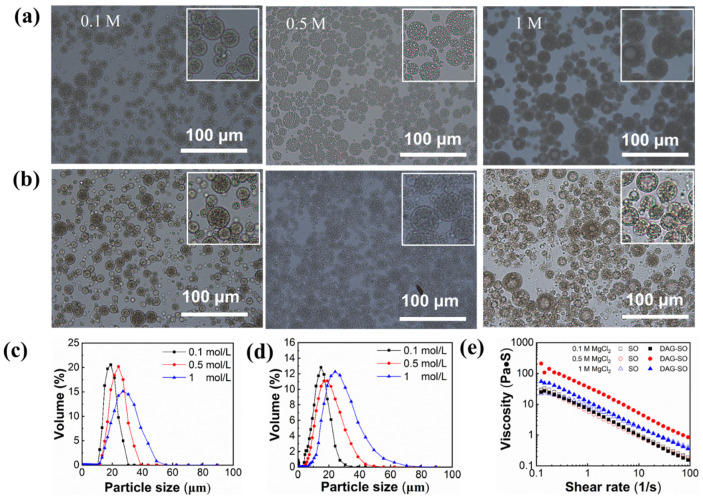
Effect of MgCl_2_ concentrations in the internal phase on the microstructure and droplet size distribution of W/O/W emulsion: SO emulsion (**a**,**c**); DAG-SO emulsion (**b**,**d**); effect of MgCl_2_ concentrations in the internal phase on the viscosity of W/O/W emulsion (**e**). The white frame indicates the magnify of the microscope images.

The influence of MgCl_2_ concentration in the inner W_1_ phase on the DAG-based W/O/W emulsion stability and the swelling phenomenon of droplets in the multiple emulsions is shown in Figure 2a,b. For samples with 0.1 M Mg^2+^, both the W/O/W emulsion and the W/O emulsion showed small droplets, indicating that the water migration in the internal and external water phases basically reached equilibrium. With 0.5 M Mg^2+^, the particle sizes of SO and DAG-SO emulsions significantly increased. The droplet size increment of the inner water phase was remarkably greater in the SO emulsion than that in DAG-SO emulsions. This indicated that the DAG-based emulsions had higher resistance to osmotic swelling due to the enhanced interfacial mechanical strength formed by the crystalline lipids. When the MgCl_2_ concentration was further increased to 1 M, the individual water droplets of the SO emulsion and DAG-SO emulsion swelled and merged into large water droplets, which was in accordance with the droplet size variation trend of the emulsions (Figure 2c,d). These results indicated that, with a high salt concentration in the W_1_ phase, significant volume expansion and coalescence of water droplets led to an increase in droplet size of W_1_ and the increase in W_1_ droplet size would result in protrusion of crystals from the “shells” and consequently increased the coalescence of emulsion globules [15]. In this case, DAG crystals were unable to prevent the diffusion of water molecules between the two phases under high osmotic pressure. 

As shown in Figure 2c,d and Table 2, emulsions with the same concentration of Mg^2+^ in the inner and outer phase (0.1 M), the mean droplet size of the emulsions containing DAG was smaller (10.86 ± 0.30 vs. 13.73 ± 0.15 µm), indicating that the presence of surface-active DAG crystals would benefit the emulsification and stabilization of water droplets. With an increasing Mg^2+^ concentration to 1 M, there was a significant increase (*p* < 0.05) in the droplet size of the SO and DAG-SO emulsions. This was because osmotic pressure imbalance would cause a faster water transfer from the W_2_ phase to the W_1_ phase, resulting in the swelling of inner water droplets [31]. Meanwhile, the size distribution of the emulsions was significantly wider with increasing Mg^2+^ concentration, which was in accordance with the microstructural change in emulsions (Figure 2a,b). 

The viscosity of W/O/W emulsions with different Mg^2+^ concentrations is shown in Figure 2e. All the emulsions displayed shear-thinning behavior. The changes in MgCl_2_ concentration in the W_1_ phase led to significant changes in the rheological properties of DAG-SO emulsion. The apparent viscosity increased with 0.5 M MgCl_2_ but reduced along with increasing concentration to 1 M. This phenomenon was consistent with the impact of salt concentration on the rheological properties of W/O emulsions in a previous report [9], which was possibly due to the transfer of water in the external phase to inner phase and possibly due to the facilitation in the adsorption density of PGPR at the water droplet interface using salt. Overall, the higher stability of DAG-containing W/O/W emulsion was attributed to the higher resistance of the oil phase to deformation and the enhanced droplet–droplet interactions with higher bridging flocculation [14].

#### 3.1.3. Effect of Osmotic Pressure

**Figure 3:**
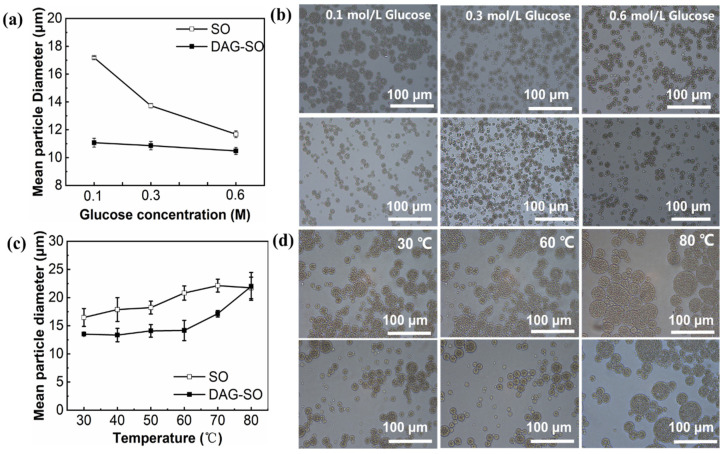
Mean particle diameter of W/O/W emulsions containing different external glucose concentrations and under different temperature treatment (**a**,**c**). Microstructures of SO- (upper row) and DAG-SO-based (bottom row) W/O/W emulsions with different external glucose concentrations and under different temperatures (**b**,**d**).

The impact of the osmotic pressure gradient on the particle diameter of the W/O droplets in the W/O/W emulsions was further evaluated by varying the glucose concentrations to the W_2_ aqueous phase to create osmotic pressure lower or higher than the continuous W_1_ phase. Initially, 0.1 M MgCl_2_ and 0.3 M glucose were added to the inner and outer water phases to keep the emulsion in osmotic pressure equilibrium. Then, the emulsion stability was investigated with varying external glucose concentration from 0.1 M to 0.6 M. As shown in Figure 3a, the droplet diameter in the DAG-SO emulsions remained constant, indicating that the sintered fat crystal shells at the W_1_/O interface and crystal network in the oil phase inhibited the osmotically driven shrinking or swelling [10,15]. Conversely, the W/O droplets in the SO emulsion whose oil phase is liquid oil swelled significantly (from 13.73 μm to 17.18 μm) when the glucose concentration was 0.1 M, and contracted (from 13.73 µm to 11.68 µm) when the glucose concentration was increased to 0.6 M, at which stage it was unable to distinguish W_1_ droplets. The results were consistent with a previous report [32], which indicated that the water phase was transferred between the external and internal phases, causing either swelling or shrinkage of the W/O droplets. This phenomenon further confirmed that DAG crystals formed a continuous network behaving as a physical barrier against osmotic pressure-driven diffusion of external and internal aqueous phases. At low external glucose concentrations, the diffusion of external water into the droplets resulted in swelling, but at high external glucose concentrations, the leakage of the internal aqueous phase droplets resulted in the shrinkage of droplets due to water loss. Hence, distinct differences exist for the double emulsions due to lipid crystallization. The microscopic images obtained for the above-mentioned emulsions (Figure 3b) were consistent with the changes in the particle size of emulsions. The size and morphology of the W_1_/O droplets in the DAG-SO emulsions were fairly similar with different external glucose concentrations. In contrast, the W_1_/O droplets in the SO-emulsions had somewhat identical morphologies when the external glucose concentration was 0.6 M, but swelled remarkably when it was reduced to 0.1 M. The microscopy results again confirmed that W/O/W emulsions containing a crystalline oil phase could have a higher resistance to osmotic pressure.

### 3.2. Effect of Heating on W/O/W Emulsion Stability

Since the lipid crystals are sensitive to the heating process, the temperature variation was carried out to evaluate emulsion stability during heating with MgCl_2_ and glucose in the inner aqueous phase at 0.1 M to keep the emulsion in a hypotonic environment. As shown in Figure 3c, the W/O droplet size of the SO emulsion was significantly larger than that in the DAG-SO emulsion at 30 °C, which was attributed to the rapid swelling of the internal droplets at elevated temperatures. When the SO emulsion was heated to 60 °C, the mean particle diameter gradually increased whereas the W/O droplets in the DAG-SO emulsion remained relatively small at 60 °C, but swelled significantly when the temperature increased to 80 °C. This was partly attributed to the melting of DAG fat crystals in the oil phase, which reduced the mechanical resistance of water molecules to migrate between the two phases. The effect of heating on the microstructure of W/O/W emulsions was shown in Figure 3d. For the SO emulsion, the W/O droplet size increased with temperature and some droplets showed significant deformation and flocculation at 80 °C. The heating process accelerated the migration of water molecules, and the fusion of W/O emulsion droplets occurred when they were close to each other and caused deformation. In contrast, the morphology of W/O droplets in DAG-SO emulsion did not change significantly at 60 °C. Still, it swelled appreciably when the temperature was raised to 80 °C due to the diffusion between the inner and outer water phases with the melting of fat crystals.

### 3.3. Effect of Gelatin in the Inner Water Phase

**Figure 4:**
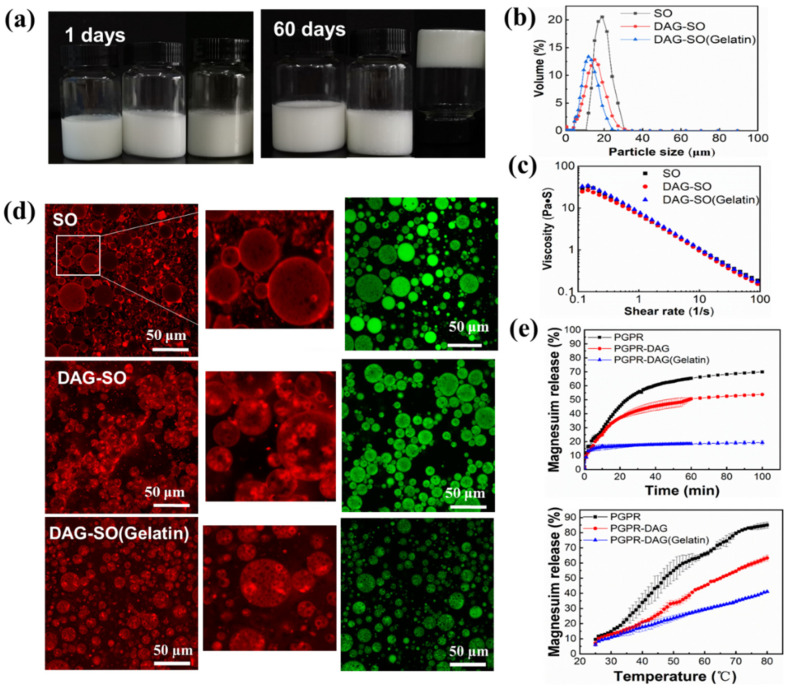
Visual appearance of PGPR, DAG-PGPR and 8 wt.% gelatin-filled DAG-PGPR W/O/W emulsions after 1 day (upper row) and 60 days (bottom row) storage at 25 °C, respectively (**a**). Particle size distribution (**b**), viscosity (**c**) and CLSM image with magnified image (**d**) of the W/O/W emulsions. Red and green color indicate the existence of lipid crystals and oil in the emulsion, respectively. Salt release from the W/O/W emulsions with alteration of time and temperature (**e**).

The effects of adding gelatin in the internal phase on the physical properties of DAG-stabilized W/O/W emulsion were further studied. All the fresh emulsions showed long-term stability during two months of storage. The physical stability was much higher than that in previous report using hydrogenated soybean oil as crystal stabilizers [14]. Therefore, the emulsion stability was largely increased by incorporating DAG as stabilizers. Among the samples, the DAG-SO (gelatin) emulsion turned into a gelation state. This was attributed to the formation of a stiff hydrogel by gelatin, which inhibited the droplet coalescence and water migration. The droplet size distribution of double emulsions are shown in Figure 4b. The droplet size of emulsion slightly decreased after adding gelatin, although the apparent viscosity of freshly prepared emulsions showed no significant difference (Figure 4b,c). The DAG crystals in the W/O/W emulsion did not influence the viscosity of the emulsion possibly because of the low proportion of oil phase (10%, *w*/*w*) in the emulsions and the high viscosity of polysaccharides present in the outer water phase [33]. Meanwhile, the gelation of the inner phase of emulsions also exerted little influence on the emulsion viscosity, although, after storage, the diffusion of water from the inner phase to the outer phase might occur and lead to gelation. Brizzi et al. [34] reported that the addition of salt ions can weaken the gelatin structure and the entanglement of polymer molecules in the presence of salt requires a longer time compared with that of the pure material, which can explain the gelation of emulsion after storage. 

To further understand the distribution of crystals and the state of the different water phases, the microstructure of emulsion was examined by confocal scanning laser microscopy (CLSM). As shown in Figure 4d, the green color stained by Nile red represents sunflower seed oil and both DAG and sodium caseinate were dyed in red with Nile blue. As can be seen in the pictures, in the outer water phase, the protein was adsorbed surrounding the oil droplets (white arrow). For the DAG-SO emulsion, the fluorescence images showed that the emulsion formed a typical three-phase (water–lipid–crystal) structure. Moreover, it was noted that the DAG crystals were packed around the inner water droplets through the Pickering stabilizing effect due to their interfacial activity and were preferentially wetted via the oil phase. This was similar to the previous report whereby glycerol monostearate, an interfacial active emulsifier with a high melting point could hinder the coalescence and collision of droplets through forming crystal layers around the droplets [16]. However, redundant crystals would protrude the droplet shells, triggering the coalescence of droplets and the sedimentation of emulsion.

Additionally, aggregated droplets can be observed possibly because of the interaction between DAG crystals at the interface, thus facilitating the droplet bridging flocculation to clusters [35]. Moreover, the crystals might pierce into the caseinate-stabilized interfacial film and thus lead to some extent of flocculation of oil droplets (Figure 4d). Notably, the droplet size of DAG-SO (Gelatin) emulsion was smaller and more uniformly distributed, and this was further proved by the droplet size distribution (Figure 4b). The presence of gelatin promoted the incorporation of more water droplets into the oil phase during the formation of W_1_/O emulsion, possibly through the solidification and emulsification effect, which reduced the aggregation of DAG crystal-coated inner water droplets. Thus, the DAG crystals at the W_1_ droplet surface and the gelled inner phase accounted for the formation of oleogel and hydrogel, enabling the formation of a multiple compartment structure with high stability in the W/O/W emulsion. This was consistent with a previous report whereby the coalescence of Pickering W/O emulsions was largely inhibited by encapsulating Pickering emulsions in hydrogels [16].

### 3.4. Salt Release Profiles

The compartmentalized nature of W/O/W emulsions would enable the more efficient encapsulation of bioactive substances. The effect of adding DAG and gelatin on the release profiles of salt ions in the inner phase of W/O/W emulsion was thus investigated. As shown in Figure 4e, the salt release rate of emulsion was fast at the first 5 min. This initial fast release might be due to the presence of unembedded Mg^2+^ in the external water phase of the double emulsion that was released after the emulsion came into contact with the bulk water phase. As time prolonged, the salt’s release rate increased, gradually achieving equilibrium after 60 min. A high osmotic pressure gradient possibly drove the quick leakage of solutes to the outer aqueous phase through an interfacial “shell” which could prevent the salt transport. Among the samples, the salt release rate of DAG-SO emulsion was lower than that of SO emulsion because DAG formed both interfacial crystal shells and crystal networks in the W/O/W emulsion. A similar result was also obtained in the recent study whereby in the W/O/W emulsions, fat crystal shells around W_1_ droplets could arrest osmosis and slow the salt leakage, whereas without fat crystals, osmotic pressure caused droplet swelling and quick salt release [36,37]. A previous report also indicated that the crystalline shell allowed a more effective protection of the globules from magnesium diffusion to the outer phase than the continuous fat network [18]. It is also noted that the emulsion with gelatin in the inner water phase showed salt release of less than 20% during the monitoring period. This was caused by the gelation of the internal aqueous phase which delayed the release of Mg^2+^.

To evaluate the effect of crystal melting on the salt release profiles, all the emulsions were heated to different temperatures. As shown in Figure 4e, the release rate of salt ions significantly increased at high temperatures for all the emulsions. The release rate of salt ions in SO emulsion showed the highest speed, and the release amount of Mg^2+^ at high temperature was significantly higher than that at ambient temperature, possibly because the heating process promoted the movement of molecules, thus accelerating the permeation and transport rate of salt ions between oil phases. For the DAG-SO emulsion, an inflection point in its salt release profile was observed at 50 °C, close to the melting point of DAG, indicating the melting of fat crystals. The DAG-SO (gelatin) emulsion also showed a slow-increasing trend as a function of temperature due to the existence of a gelled inner phase. The presence of protein in the W_1_ phase can improve the stability of the double emulsion and control the release rate of encapsulated solutes in the double emulsion. This was in accordance with previous reports whereby the protein in the inner phase can slow down the release of Mg^2+^ in the multiple emulsion, and improve its textural profiles [24].

### 3.5. Encapsulation Efficiency (EE) of EGCG and Curcumin in the W/O/W Emulsions

**Figure 5:**
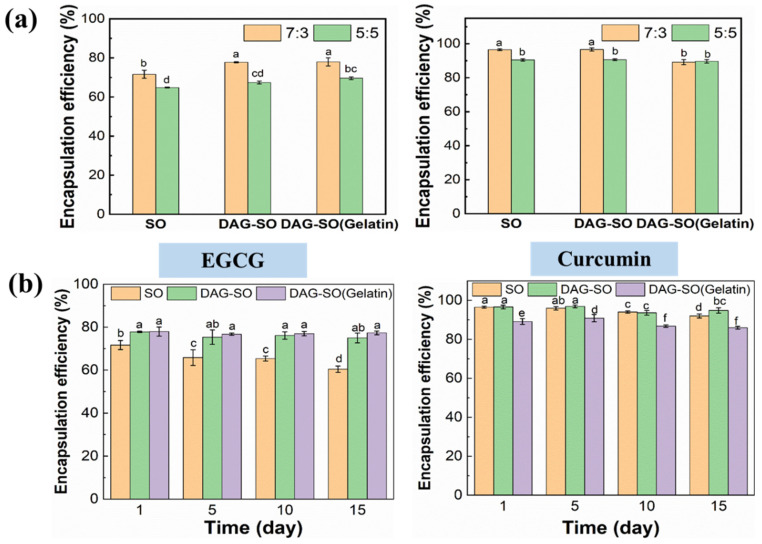
The encapsulation efficiency of EGCG and curcumin in W/O/W emulsions with different W_1_/O ratios (**a**). The encapsulation efficiency of EGCG and curcumin in different W/O/W emulsions (**b**). Different letters (a–e) on the columns indicate significant differences between samples (*p* < 0.05).

The encapsulation efficiency of EGCG and curcumin in double emulsions with different W_1_:O ratios is shown in Figure 5a. The emulsion with a W_1_:O ratio of 7:3 showed higher EE than that with the ratio of 5:5. This indicated that the inner water phase content is critical in determining the emulsion stability. With more W_1_ phase incorporation, the W_1_/O emulsion can better retain EGCG. For the emulsions with different formulations, the presence of solid lipid DAG significantly increased the retention efficiency EGCG from ~60% to ~75% during the 15 days of storage (*p* < 0.05), indicating that the presence of a crystal layer surrounding the inner water droplet can efficiently prevent the loss of the water-soluble bioactive in the inner aqueous phase by restricting the leakage of inner water. The retention value was similar compared with previous study which indicated that the DAG crystal-coated water droplet can well maintain the encapsulation yield of EGCG in the double emulsion [7]. Nonetheless, the samples with gelatin slightly reduced the EE of curcumin compared to the SO and DAG-SO emulsions which might be because the interaction between gelatin and DAG caused there to be less DAG crystals surrounding the oil droplets and thus a lower protection ability for the oil-soluble compounds [7].

### 3.6. In Vitro Digestion Profile of W/O/W Emulsions

**Figure 6:**
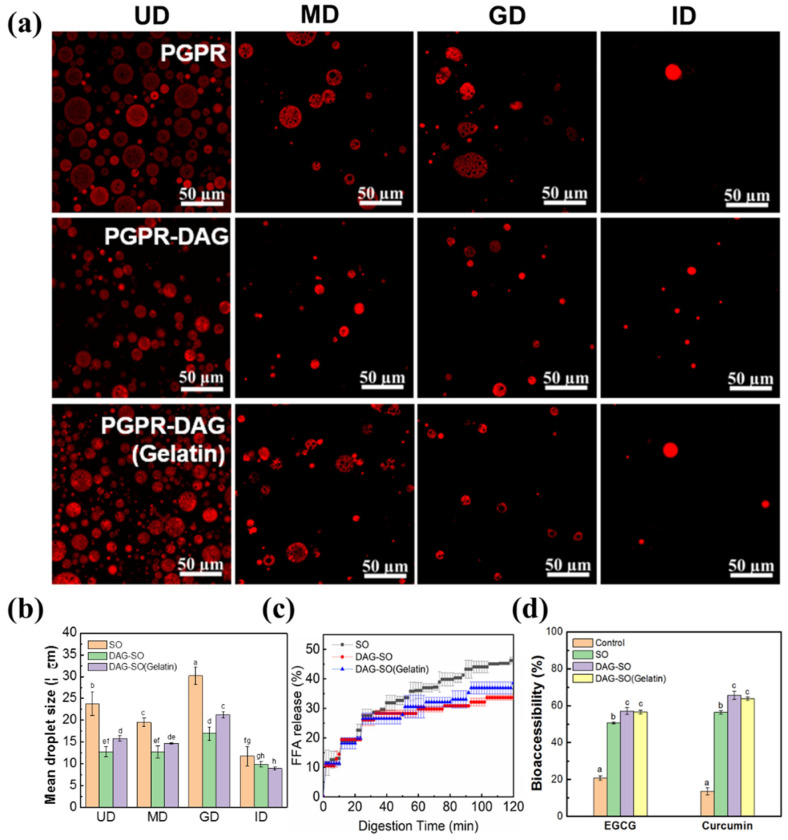
CLSM images of PGPR, PGPR-DAG and PGPR-DAG (Gelatin)-based W/O/W emulsions after simulated digestion. (UD = undigested; MD = mouth digestion; GD = gastric digestion; ID = intestinal digestion) (**a**). The red color indicates the presence of oil in emulsions. Mean particle diameter (**b**) and release of free fatty acid from emulsions vs. digestion time (**c**). Bioaccessibility of EGCG and curcumin after simulated gastrointestinal digestion (**d**). Different letters (a–h) on the columns indicate significant differences (*p* < 0.05) between samples.

The oil droplets’ diameter and microstructure of the emulsions in simulated gastric and intestinal phases were monitored. As shown in Figure 6, at the beginning of the digestion process, the presence of DAG in the oil droplets significantly influenced the emulsions’ particle size, leading to a smaller O/W_2_ droplet size (Figure 6a,b). The microstructure of SO and DAG-SO (gelatin) emulsions showed an intact water-in-oil-in-water structure with no expansion of droplet volume in the mouth phase. After simulated gastric digestion of emulsions, the droplet size was rarely changed from the range of 15~22 µm. In contrast, the SO emulsion showed evident droplet deformation after the gastric phase with more significantly increased droplet size (Figure 6b). Thus, the emulsions with the coexistence of DAG and PGPR as oil droplet stabilizers showed high stability under gastric conditions due to the formation of stronger interfacial crystal shells. 

The W/O/W emulsions’ droplet size decreased after simulated small-intestine digestion, but the double-emulsion structure was destroyed (Figure 6a). A total release of W_1_-droplets was observed but the oil droplets were still present in the W_2_ phase. The structural changes and the destruction of inner W_1_-droplets during in vitro digestion was possibly caused by the diffusion or coalescence of the W_1_-droplets with the W_2_-phase. In addition, it was observed that, after intestinal digestion, the droplet size was alike for different emulsions, which indicated that the double emulsions were prone to coalescence with the presence of pancreatic lipases or bile salts with surface activity. These compounds further acted as substituents for original surfactants. 

The influence of the DAG and internal gelatin on the lipid digestibility of the emulsions was further assessed by FFA release (%) after digestion. It is displayed in Figure 6c that all the emulsions showed a brief delay in the FFA release ratio at the beginning of digestion stage, and then showed a steep increase in release amount. The gradual increase in bioactive compound content indicated that the emulsions effectively provided sustained release under oral and gastrointestinal conditions [7]. The final FFA release largely depended on the emulsion formulation, whereby SO emulsion showed the highest lipid digestion extent, achieving ~45% of FFA release after 2 h digestion. In contrast, the DAG-SO emulsion showed a lower degree of hydrolysis, with FFA values of 32% at the end of digestion. DAG-SO (gelation) showed a similar digestion profile, although the final FFA value was slightly higher. Therefore, the presence of lipid crystals caused an interfacial impediment to reduce the lipid digestion process of emulsion since lipid digestion mainly occurred at the interfacial area involving binding of lipase onto the droplet surface of emulsion. This also indicated that the high melting point of DAG and the modified interfacial structure of emulsion by DAG were the main factors affecting the digestion process since the viscosity of these emulsions showed no significant difference (Figure 4c). Moreover, the oil droplet flocculation observed in the DAG-SO emulsion (Figure 4d) also caused a physical impediment for lipid digestion, and the gelation incorporation promoted more uniformly distributed oil droplets and thus slightly increased the lipid digestion degree. Overall, the release profiles were close to the results previously reported by Velderrain-Rodriguez at al. [35]; they observed that the FFA release values in the carboxymethyl cellulose–Tween 20-stabilized double emulsion achieved 40% due to the high emulsion viscosity. 

As reported, the bioaccessibility of curcumin is related to a micellization ability during digestion [5]. As shown in Figure 6d, compared with that of bioactives encapsulated in the control (water or bulk oil), after digestion, the concentration of EGCG/curcumin in the water and micelle phase was much higher for the emulsions in the presence of DAG. This was partly attributed to the higher EE of these two compounds in the double emulsion (Figure 5). Simultaneously, the emulsifying properties of DAG and higher fat content in the DAG-SO emulsion might facilitate the formation of micellarization and enable the greater solubility of curcumin in the micelle phase, which increases its bioaccessibility [38,39]. The bioaccessibility of bioactives was higher compared with previous research using whey protein and konjac glucomannan as stabilizers for W/O/W emulsion, which indicated that the presence of DAG is suitable for application in double emulsions as crystal stabilizers for the enhancement of the bioavailability of water-soluble and lipid-soluble compounds [40].

## 4. Conclusions

W/O/W emulsions with a multi-compartmentalized structure were fabricated and the stabilization effects of DAG in the oil phase were highlighted in this study. The 6 wt.% DAG crystals adsorbed around the W_1_ droplets acted as effective Pickering stabilizers to increase the emulsion stability and can restrict the water transfer between the W_1_ and W_2_ phases. The emulsion stability against osmotic pressure and heating treatment was greater than the regular SO-based W/O/W emulsion because of the interfacial crystalline shell and oil phase’s mechanical strength. Moreover, the presence of 1 wt.% gelatin in the inner phase can efficiently reduce the salt release rate. The DAG-based double emulsion showed a higher encapsulation efficiency and retention of EGCG during storage. The W/O/W emulsion showed a reduced simulated digestion degree but higher bioaccessibility of curcumin and EGCG due to a better micellization of the digested bioactives. The results in this study are meaningful for the fabrication of functional cargo delivery systems with long-term physical stability, slow release rate and high bioaccessibility.

## Figures and Tables

**Table 1:** Mean droplet size of W/O/W emulsions with different PGPR concentration and W1:O ratios.

Samples	PGPR (*w*/*w*)			W1:O Ratios		
	1%	3%	5%	3:7	5:5	7:3
Mean droplet size (μm)	14.71 ± 0.40	13.63 ± 0.42	10.53 ± 0.28	5.53 ± 0.12	9.86 ± 0.15	10.86 ± 0.30

**Table 2:** Mean droplet size of W/O/W emulsions with different MgCl_2_ concentrations.

MgCl_2_ Concentration		0.1 M	0.5 M	1 M
Mean droplet size (μm)	SO	13.73 ± 0.15	21.18 ± 0.10	24.30 ± 0.13
	DAG-SO	10.86 ± 0.30	15.87 ± 0.20	22.73 ± 0.17

## Data Availability

Data are contained within the article.
